# Mucilage Polysaccharide Composition and Exudation in Maize From Contrasting Climatic Regions

**DOI:** 10.3389/fpls.2020.587610

**Published:** 2020-12-08

**Authors:** Meisam Nazari, Sophie Riebeling, Callum C. Banfield, Asegidew Akale, Margherita Crosta, Kyle Mason-Jones, Michaela A. Dippold, Mutez Ali Ahmed

**Affiliations:** ^1^Division of Biogeochemistry of Agroecosystems, Georg-August University of Göttingen, Göttingen, Germany; ^2^Chair of Soil Physics, Bayreuth Center of Ecology and Environmental Research (BayCEER), University of Bayreuth, Bayreuth, Germany; ^3^Department of Terrestrial Ecology, Netherlands Institute of Ecology (NIOO-KNAW), Wageningen, Netherlands

**Keywords:** agroecological zones, genotype, maize, mucilage, root exudation, vapor pressure deficit

## Abstract

Mucilage, a gelatinous substance comprising mostly polysaccharides, is exuded by maize nodal and underground root tips. Although mucilage provides several benefits for rhizosphere functions, studies on the variation in mucilage amounts and its polysaccharide composition between genotypes are still lacking. In this study, eight maize (*Zea mays* L.) genotypes from different globally distributed agroecological zones were grown under identical abiotic conditions in a randomized field experiment. Mucilage exudation amount, neutral sugars and uronic acids were quantified. Galactose (∼39–42%), fucose (∼22–30%), mannose (∼11–14%), and arabinose (∼8–11%) were the major neutral sugars in nodal root mucilage. Xylose (∼1–4%), and glucose (∼1–4%) occurred only in minor proportions. Glucuronic acid (∼3–5%) was the only uronic acid detected. The polysaccharide composition differed significantly between maize genotypes. Mucilage exudation was 135 and 125% higher in the Indian (900 M Gold) and Kenyan (DH 02) genotypes than in the central European genotypes, respectively. Mucilage exudation was positively associated with the vapor pressure deficit of the genotypes’ agroecological zone. The results indicate that selection for environments with high vapor pressure deficit may favor higher mucilage exudation, possibly because mucilage can delay the onset of hydraulic failure during periods of high vapor pressure deficit. Genotypes from semi-arid climates might offer sources of genetic material for beneficial mucilage traits.

## Introduction

Plant roots have intensive physical, chemical and biological interactions with the surrounding soil (Haichar et al., 2008; Micallef et al., 2009; Bender et al., 2016; Leff et al., 2018). Roots exude a diverse range of metabolites that modify the physical and chemical environment of the rhizosphere (Hinsinger et al., 2009; Carminati and Vetterlein, 2013; Oleghe et al., 2017) and mediate plant-plant and plant-microbe interactions (Badri and Vivanco, 2009; Dutta et al., 2013; Williams and de Vries, 2020).

Plants exude about 25% of their total photosynthetic output into the rhizosphere, of which approximately half is in the form of mucilage (Chaboud, 1983; Walker et al., 2003). Mucilage is a gelatinous high-molecular-weight substance produced by almost all plants (Ahmed et al., 2014). Maize (*Zea mays* L.) exudes mucilage both from its nodal and underground roots. Maize nodal root mucilage has very similar properties to its underground root mucilage (Chaboud, 1983; Osborn et al., 1999). Mucilage produced by the nodal roots of some maize landraces harbors nitrogen-fixing bacteria, contributing to the fixation of 29% to 82% of the plant’s nitrogen nutrition (Van Deynze et al., 2018; Amicucci et al., 2019; Bennett et al., 2020). This nitrogen fixation can increase maize yield and nitrogen use efficiency, especially in regions where agriculture suffers from poor soil fertility. Moreover, mucilage has been shown to increase the rhizosphere water content (Young, 1995; Carminati et al., 2010; Naveed et al., 2019), attenuate the gradients in soil water potential, and thereby facilitate root water uptake during soil drying (Ahmed et al., 2014). However, mucilage becomes water repellent upon drying (Ahmed et al., 2015; Zickenrott et al., 2016) and delays rewetting of the rhizosphere (Zarebanadkouki et al., 2018). Mucilage has also been implicated in reducing friction against the growing root (Iijima et al., 2003), ameliorating aluminum toxicity (Horst, 1995), and stabilizing soil aggregates (Czarnes et al., 2000). Mucilage is also a substrate for microbial decomposition (Mary et al., 1993; Ahmed et al., 2018a) and can provide a unique microbial habitat (Ahmed et al., 2018c). Mucilage likely contributes to plant resistance to water stress, and may be particularly important in water-limited environments (Ahmed et al., 2018b), and those with high vapor pressure deficit (VPD), where mucilage may delay the onset of hydraulic failure during drought (Grossiord et al., 2020).

Mucilage is mainly composed of polysaccharides but also contains proteins, minerals, and lipids (Carminati and Vetterlein, 2013; Koocheki et al., 2013; Alizadeh Behbahani et al., 2017). Though the monosaccharide composition and polysaccharide structure of mucilage has been previously studied, only recently have the first links been made between mucilage chemistry and its functional properties (Amicucci et al., 2019). This work has shown that mucilage chemical composition has important implications for the structure of colonizing microbial communities (Amicucci et al., 2019). Elucidating the polysaccharide composition of mucilage will therefore be a crucial step for understanding mucilage functions and the interaction of roots with their environment. Previous efforts to elucidate the mucilage composition of maize nodal roots have revealed that this mucilage is rich in fucose, galactose, and arabinose (Van Deynze et al., 2018; Amicucci et al., 2019). However, the observed similarity in the mucilage composition of these previous studies could be attributed to the use the same maize landrace, Sierra Mixe, Mexico (*Zea mays* Y.). Thus, similarities or differences in mucilage composition between maize genotypes remain unknown. Current knowledge indicates that the root exudates of different genotypes of the same plant species can differ in composition (Bulgarelli et al., 2015; Iannucci et al., 2017). However, these results largely consider low molecular weight organic compounds, which are rapidly consumed by soil microbial communities (Gunina and Kuzyakov, 2015). In contrast, the longer mean resistance time of mucilage in the rhizosphere (Mary et al., 1993) suggests that it can have greater spatial and temporal impact on root-soil interactions (Ahmed et al., 2018a).

Plant breeding has mainly focused on aboveground traits while only a few attempts have targeted root traits that support plant-soil interactions (Lammerts Van Bueren et al., 2011). Our knowledge of the genetic basis for the amount and composition of mucilage exudation is limited (Monchgesang et al., 2016), hindering the exploitation of this trait for breeding purposes (Kuijken et al., 2015; Fernández-Aparicio et al., 2016). Thus, an important research task is to identify the genetic basis of mucilage exudation and polysaccharide composition in support of future maize breeding and agricultural sustainability (Bennett et al., 2020). Addressing this issue requires disentangling a complex set of factors including different plant genotypes and growth conditions (Micallef et al., 2009). The first step of such an approach—testing different plant genotypes under constant soil and climatic conditions—is the scope of this study.

The aims of this study were to quantity and characterize the nodal root mucilage of eight maize genotypes from different agroecological zones grown under the same abiotic and biotic conditions. To characterize the mucilage, neutral sugar and uronic acid compositions were analyzed by gas chromatography-mass spectrometry (GC-MS). The mucilage exudation amount was measured for quantitative assessment. We hypothesized that mucilage polysaccharide composition and exudation amount are adapted to the agroecological zone in which a variety was bred, with VPD being a key variable controlling mucilage composition and amount. Thus, these genetically inherited traits will be evident even if these genotypes are grown under identical abiotic environmental conditions in a field experiment.

## Materials and Methods

### Field Experiment

The field experiment was performed as a randomized complete block design consisting of three replicates of each genotype at the teaching facility “Landwirtschaftliche Lehranstalten” near Bayreuth, Bavaria, Germany (49° 55′ 47″ North, 11° 33′ 8″ East and 344 m above sea level). The soil was classified as loamy silt with 67% sand, 11% silt, and 22% clay. Bayreuth has a temperate climate with mean annual temperature and annual precipitation of 8.3°C and 638 mm, respectively. The maximum temperature during the 2019 maize season was 36.76°C on 25th of July (daily average: 26.24°C) and the minimum temperature was −4.98°C on 12th of April (daily average: 1.29°C). Minimum precipitation was zero mm per day with nearly rain-free periods beginning of April and end of May until first half of June. The maximum rainfall was on 7th of August with 22.91 mm. The VPD for Bayreuth during the maize growing period was 0.19 kPa.

The maize genotypes were sown manually in the first week of May 2019 in strips of 0.2 × 9 m with 75 cm row distance between the strips. On each side of the strip, there were three rows of plants of a commercial maize genotype to avoid border effects. The row of each genotype was replicated three times (*n* = 3). Weeds were manually removed but no fertilizers or pesticides were applied. Due to the hot weather and absence of rain in June, we applied irrigation for the whole experiment. Mucilage replicates were sampled from each of the three rows (*n* = 3) at the end of tassel emergence (BBCH 59). To gain a sufficient amount of mucilage for follow up analysis, several plants per row were sampled randomly.

### Genotypes of Maize

Eight maize genotypes from contrasting agroecological zones of the world were used (Table 1). The genotype Kentos is a silage maize developed by the breeding company KWS SAAT SE & Co., KGaA (Einbeck, Germany) for the German market. Germany is classified as a cool temperate climate. The genotype KXB 8383 is also produced and marketed by the French branch of the KWS company as grain maize for distribution in France. While suited to a temperate climate, this genotype is also tolerant to the Mediterranean climate in the south of the country. The Italian branch of KWS produces the genotype Kerubino at a breeding station near Venice. The climate there is warm temperate. Keravnos is a Turkish maize genotype produced by KWS Türk. Turkey has a Mediterranean climate with hot, dry summers. The genotypes DH 02 and DH 04 are produced by the Kenya Seed company (Kitale, Kenya). The genotype DH 02 comes from dryland regions in the southeast of the country (annual precipitation < 700 mm). The genotype DH 04 is bred for the lowland regions where climate is tropical (annual precipitation < 900 mm). The Indian 900 M Gold and 30 V 92 genotypes are produced by Dekalb and Pioneer Hi-Bred companies, respectively. Both companies have their breeding station in Hyderabad, India, with a semi-arid and very hot steppe climate (annual precipitation < 800 mm).

**TABLE 1:** Information on the maize genotypes, their agroecological zones, and breeding methods.

Region	Country	Climate	Temperature (°C)^*a*^	Relative humidity (%)^*a*^	*P* (mm)^*b*^	Genotype	Breeding method
Central Europe	Germany	Temperate	15.8	77.7	132–560	Kentos	Hybrid
	France	Temperate	15.7	60	105–660	KXB 8383	Hybrid
Southern Europe	Turkey	Mediterranean	16.4	59	86–332	Keravnos	Hybrid
	Italy	Mediterranean	18.4	72.5	240–1,100	Kerubino	Hybrid
Africa	Kenya	Semi-arid	24.8	75	62–720	DH 02	Hybrid
	Kenya	Tropical	24.6	83	62–867	DH 04	Hybrid
Asia	India	Semi-arid	25.7	72.3	324–2,044	900 M Gold	Hybrid
	India	Semi-arid	25.7	72.3	324–2,044	30 V 92	Hybrid

*^*a*^Temperature and relative humidity are the mean for the maize growing season in the related agroecological zone within the past 10 years. ^*b*^P (precipitation) is the sum over the entire growing season as range minimum to maximum of observed precipitation within the past 10 years.*

### Sampling of Mucilage

**FIGURE 1:**
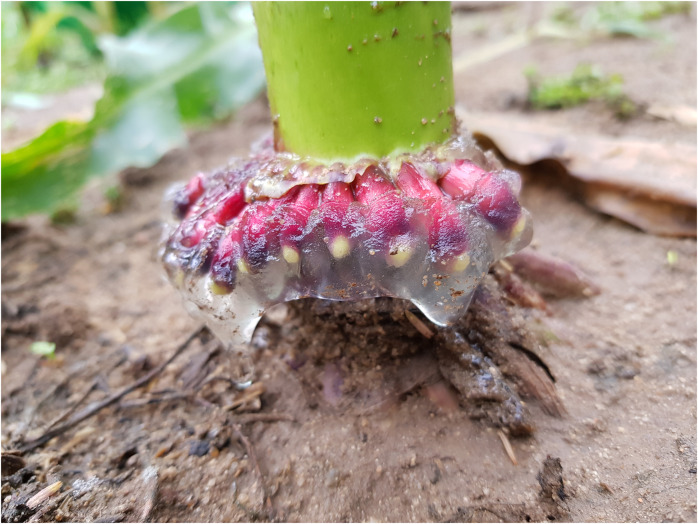
Mucilage secreted by nodal roots of the maize genotype Kerubino at the end of tassel emergence after a rainfall event.

Mucilage samples were taken at the end of tassel emergence (BBCH 59) from each of the three rows (*n* = 3) using the method of Ahmed et al. (2015). Maize second and third nodal roots covered in mucilage were selected (Figure 1). The nodal roots were cut from the stem and placed in aluminum trays. In the laboratory, soil and plant residues on the nodal roots were removed by distilled water in a coarse sieve. Thereafter, the mucilage-covered roots were submerged in distilled water until the mucilage was fully water-saturated. After 1 day, the excess water was discarded through a sieve with a mesh size of 500 μm. The hydrated mucilage was aspirated from the nodal roots with syringes. The remaining mucilage on the root tip was removed with fine forceps. The mucilage was collected in 20 ml vials, frozen at −18°C, and subsequently freeze-dried (Beta 1-8 LSCplus, Christ, Osterode, Germany).

### Preparation of Samples

Freeze-dried mucilage (20 mg) was weighed into centrifuge tubes. Lipids were sequentially pre-extracted by 20 ml methanol, followed by 20 ml dichloromethane:methanol (1:1, v:v) and by 20 ml dichloromethane. The pre-extractions were performed in an ultrasonic bath (35 kHz, 25°C, 80 W nominal power, Bandelin Sonorex RK100, Berlin, Germany) for 10 min, followed by centrifugation at 1,500 × g for 10 min. The pellet was dried under nitrogen gas and weighed. Only chemicals of p.a. grade or better were used for the subsequent analyses. Analysis follows in its principles the method of Zhang and Amelung (1996).

For neutral sugar and uronic acid analyses, 10 ml 4 M trifluoroacetic acid (TFA) was added to each sample, capped and hydrolyzed at 105°C for 8 h. The hydrolysate was filtered through glass fiber filters (GF6, Whatman GmbH, GE Healthcare, Freiburg, Germany) with 5 ml of ultrapure water, and the first internal standard (0.05 mg allose) was added. Thereafter, the sample was dried in a rotary evaporator at 40°C and 30 mbar. To completely remove TFA residues, 0.5 ml of water was added to the conical flask and evaporated again. This step was repeated once more. Samples were transferred to reaction vessels with three times 0.5 ml of water and dried under pure nitrogen gas. To split the samples into two parts for measurement of neutral sugars and uronic acids, they were re-dissolved in 500 μl of water, sonicated for 10 min in the ultrasonic bath, and then an aliquot of 250 μl was transferred to new reaction vessels. The samples were evaporated again and stored at −20°C prior to derivatization.

### Derivatization and Measurement of Neutral Sugars

Seven volumes (ranging from 10 to 800 μl) of an external standard stock solution of 0.5 mg ml^–1^ of allose (D +), arabinose (D−), fucose (L−), galactose (D +), glucose (D +), mannose (D +), rhamnose (L +), ribose (D−), and xylose (D +), were pipetted into reaction vessels and dried under a nitrogen gas stream. Samples and external standards were derivatized in a two-step procedure to aldononitrile acetates. First, 300 μl of hydroxylamine hydrochloride and 4-(dimethylamine) pyridine (32 mg ml^–1^) dissolved in pyridine-methanol (4:1, v:v) was added to the samples and standards, capped and heated to 80°C for 30 min. After cooling down, 1 ml acetic anhydride was added to all vessels, which were then heated again to 80°C for 30 min, following the procedure of Zhang and Amelung (1996). After the derivatization, 15 μg of the second internal standard (IS2) methyl tridecanoate was added to derivatized and purified samples (filtered through anhydrous Na_2_SO_4_) and standards and they were transferred with a mixture of ethyl acetate/n-hexane (1:1, v:v) to 300 μl inserts in GC vials.

The analytes were separated by gas chromatography (Agilent 7820A GC, Agilent Technologies, Waldbronn, Germany) and detected by mass spectrometry (Agilent 5977B, Agilent Waldbronn, Germany). Helium with a constant flow rate of 1.1 ml min^–1^ was used as the carrier gas through an OV 17-MS capillary column (Macherey Nagel, Düren, Germany, 30 m length, 250 μm inner diameter, 0.25 μm film thickness). An aliquot of 1 μl per sample was injected with a split ratio of 30:1 at an injector temperature of 250°C. The oven program started at 100°C, held for 1 min, then heated by 20°C min^–1^ to 175°C and held at this temperature for 3 min. The temperature was increased further by 4°C min^–1^ to 235°C, held for 3 min and finally raised to 300°C at 50°C min^–1^ and held for 7 min. Further detailed GC parameters can be found in the Supplementary Materials to Banfield et al. (2018). The mass-sensitive detector was run in scan mode (50–550 amu) with electron ionization energy of 70 eV.

### Derivatization and Measurement of Uronic Acids

Eight volumes (ranging from 10 to 800 μl) of an external standard stock solution containing 1 mg ml^–1^ galacturonic acid, glucuronic acid and 3-O-methylglucose were pipetted into reaction vessels and treated as samples. 0.05 mg of 3-O-methylglucose were added to the samples as an internal standard. Derivatization was performed in two steps: First, 200 μl NMP was added as a solvent and 200 μl methoxyamine hydrochloride solution (20 mg ml^–1^ pyridine) was used for methyloxime formation. Vessels were heated to 75°C for 30 min. Thereafter, 400 μl N,O-bis(trimethylsilyl)trifluoroacetamide was applied for silylation of the hydroxyl groups to trimethylsilyl groups. Vessels were heated for 5 min at 75°C. Details were as described by Banfield et al. (2018). After derivatization, 50 μg of hexadecane were added as the IS2 and the samples were transferred, including their derivatization reagents, into GC vials for measurement within 8 h of derivatization.

The analytes were separated by gas chromatography (Agilent 7890 GC, Agilent Waldbronn, Germany) and detected in a mass-sensitive detector (Agilent 7000B Triple Quadrupole MS, Agilent Waldbronn, Germany). The GC was equipped with a DB-5MS column (30 m length, 250 μm inner diameter, 0.25 μm film thickness). Helium with a flow rate of 1.5 ml min^–1^ was used as the carrier gas. An aliquot of 1 μl was injected at 250°C and 0.68 bar at a split ratio 50:1. The oven program started at 145°C, held for 0.5 min and then heated to 160 at 10°C min^–1^, held again for 0.5 min and heated at 6°C min^–1^ to 185°C. The temperature was raised to 185°C at a rate of 6°C min^–1^, held for 0.5 min and increased to 300°C at 100°C min^–1^. The detector was set to scan mode (all fragments from 50 to 550 amu) and electron ionization energy of 70 eV.

### Integration and Quantification of Sugars and Uronic Acids

Total ion current chromatogram peaks were integrated with the Agilent Mass Hunter Quantitative Data Analysis software (Agilent Technologies, Waldbronn, Germany), always ensuring peak identity by comparison of characteristic fragments with the external standards. Analyte peak areas were normalized to the peak areas of the sample’s respective IS2 peak (methyl tridecanoate, hexadecane). Quantification was performed based on a linear regression of the external standards’ peak areas to the external standard amounts. Furthermore, a recovery correction using IS1 provided absolute quantification of polysaccharide-derived monosaccharide content.

### Measurement of Mucilage Exudation Amount and Saturation Water Content

Mucilage exudation amount was expressed as dry mass of collected mucilage per unit area of the nodal root surface (g mm^–2^). The root surface was estimated using the formula for the surface of a cylinder A = 2π × r × h. The diameter and length of the roots were measured with calipers. In addition, the saturation water content of mucilage was calculated by the subtraction of the mucilage wet weight from dry weight divided by its dry weight. The calculation of mucilage exudation and saturation water content was carried out with the average of 10 nodal roots per genotype.

### Calculation of Vapor Pressure Deficit

The following equation was used to calculate VPD during the growing season of maize for each agroecological zone (Marengo et al., 2008; Seager et al., 2015):

(1) $$V\,P\,D\,\left(k\,P\,a\right)=0.611\times e\,x\,p\,\left(\frac{17.5\times T}{240.987+T}\right)-0.611\times e\,x\,p\,\left(\frac{17.5\times T_{d}}{240.987+T_{d}}\right)$$

Where, T is the mean temperature (°C) and T_*d*_ is the dew point (°C).

T and relative humidity (RH%) were used to calculate the T_*d*_:

(2) $$T_{d}=\frac{243.04\times \left(l\,n\,\left(\frac{R\,H}{100}\right)+\frac{17.625\times T}{243.04+T}\right)}{17.625-\left(l\,n\,\left(\frac{R\,H}{100}\right)+\frac{17.625\times T}{243.04+T}\right)}$$

It is noted that T and RH were the mean for the maize growing season in each agroecological zone.

### Statistical Analysis

The statistical design follows a randomized block design with blocks expressed as rows in our experiment and each block serving as one of the field replicates. Each block contained one row with each genotype representing the levels of our treatments. All data were analyzed by SPSS 25 (SPSS Inc., Chicago, IL, United States). The data were tested for homogeneity of variance by Levene’s test and normality by visual inspection of Q-Q plots, respectively, and transformed appropriately if they did not meet these prerequisites. One-way analysis of variance (ANOVA) was used to test for significant differences of the means between the genotypes at a significance level (α) of 0.05. Tukey’s HSD (Honestly Significant Difference) test was used for pair-wise comparison of the arithmetic means. Linear regression (α = 0.05) was used to detect associations between the genotypes’ mucilage exudation amount and also saturation water content and the VPD of their agroecological zones. Linear regression was also used to find associations between polysaccharide composition and VPD. Multiple regression (α = 0.05) was used to identify correlations between the mucilage saturation water content and its mucilage polysaccharide composition.

## Results

### Mucilage Polysaccharide Composition

**FIGURE 2:**
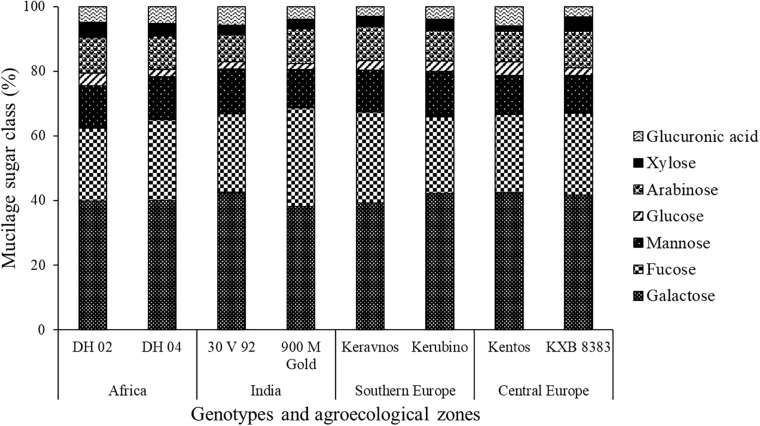
Proportion of the neutral monosaccharides and uronic acids in the nodal root mucilage of the maize genotypes. Displayed is the proportion of the individual monosaccharides or uronic acids as proportion of the total monosaccharide and uronic acid content of the respective mucilage sample. Values represent averages of three field replicates (*n* = 3).

**FIGURE 3:**
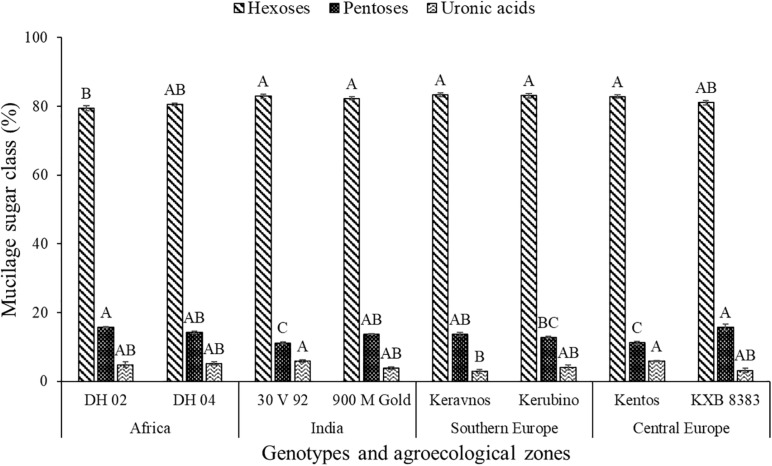
Proportion of the sugar compound classes in the nodal root mucilage of the maize genotypes. Different letters on each bar show a statistically significant difference (Tukey’s HSD, at *P* ≤ 0.05). Error bars indicate the standard error of the mean (*n* = 3).

**FIGURE 4:**
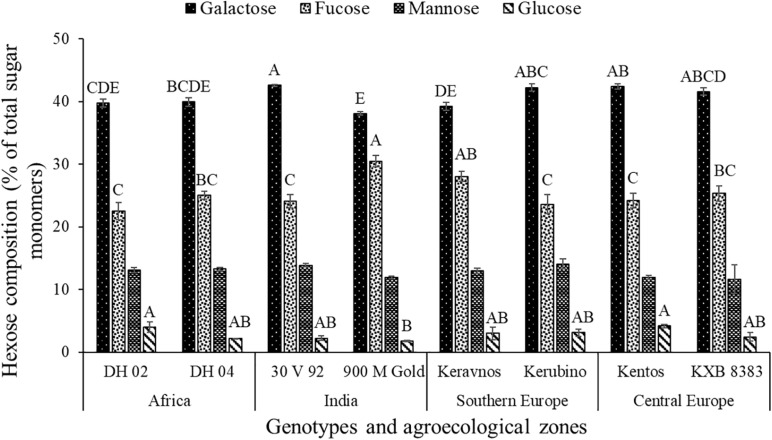
Proportion of hexose monosaccharides in the nodal root mucilage of the maize genotypes. Different letters on each bar show a statistically significant difference (Tukey’s HSD, at *P* ≤ 0.05). Error bars indicate the standard error of the mean (*n* = 3).

The hexoses galactose (∼39–42%), fucose (∼22–30%), mannose (∼11–14%), and glucose (∼1–4%) were found in the nodal root mucilage of all maize genotypes (Figure 2). The hexose rhamnose was below the limit of detection. With exception of mannose and rhamnose, there were significant differences in hexose composition between the maize genotypes (at *P* ≤ 0.05, Table 2). The Kenyan genotype DH 02 had the lowest overall proportion of hexoses, whereas 3.5% higher hexose proportions were found in the genotypes 30 V 92, 900 M Gold, Keravnos, Kerubino, and Kentos (at *P* ≤ 0.05, Figure 3). The Indian genotypes had the highest sum of galactose and fucose, the two most abundant monomers, but showed significantly contrasting partitioning of these two monomers (at *P* ≤ 0.05, Figure 4). The genotype 30 V 92 had the highest proportion of galactose (42.6%) and the lowest proportion of fucose (24.1%), whereas vice versa 900 M Gold displayed highest fucose (30.2%) and lowest galactose (38.1%) proportions within the set of studied genotypes. Besides this, there is generally a remarkable similarity in the proportion of galactose and fucose and only minor differences in distribution of other hexose monomers were observed. Kentos and DH 02 had the highest (4.2 and 3.9%, respectively) and 900 M Gold the lowest proportion of glucose (1.8%) (Figure 4).

**TABLE 2:** Analysis of variance for the mucilage sugar composition, exudation, and saturation water content of the maize genotypes (one-way ANOVA, at *P* ≤ 0.05).

Sources of variation	df	Sum of squares	Mean square	*F*	*P*-value
Hexoses	7	42.66	6.09	6.09	<0.001*
Pentoses	7	0.06	0.01	16.64	<0.0001*
Glucuronic acid	7	27.54	3.93	4.06	<0.01*
Galactose	7	60.19	8.59	10.37	<0.0001*
Fucose	7	142.63	20.33	11.59	<0.0001*
Mannose	7	0.39	0.04	2.59	NS
Glucose	7	0.35	0.05	4.68	<0.01*
Arabinose	7	0.04	0.006	11.70	<0.0001*
Xylose	7	0.33	0.04	10.19	<0.0001*
Mucilage exudation	7	1.87	0.26	8.06	<0.0001*
Mucilage saturation water content	7	123058.57	17579.79	4.01	<0.01*

*, significant; NS, not significant.

**FIGURE 5:**
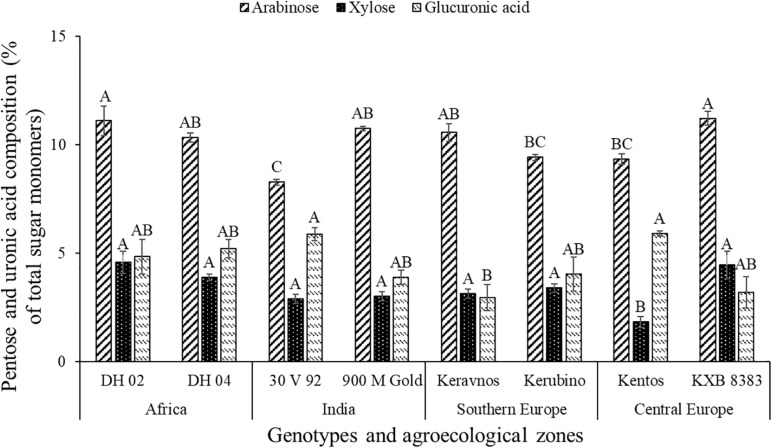
Proportion of pentose monosaccharides and glucuronic acid in the nodal root mucilage of the maize genotypes. Different letters on each bar show a statistically significant difference (Tukey’s HSD, at *P* ≤ 0.05). Error bars indicate the standard error of the mean (*n* = 3).

The pentoses comprised arabinose (∼8–11%) and xylose (∼1–4%), whereas ribose was below the limit of detection (Figure 2). The maize genotypes significantly differed in pentose composition (at *P* ≤ 0.05, Table 2). Kentos and 30 V 92 had the lowest proportion of pentoses (4.5% less than DH 02 and KXB 8383, at *P* ≤ 0.05, Figure 3). In the case of 30 V 92 this was due to a 2.9% lower arabinose proportion and in the case of Kentos by 2.7% lower xylose proportion relative to DH 02 or KXB 8383 (at *P* ≤ 0.05, Figure 5). The Kenyan genotype DH 02 and the central European genotype KXB 8383 had the highest arabinose proportions. Glucuronic acid (∼3–5%) was the only uronic acid detected. The proportion of glucuronic acid of the total sugar monomers was significantly different among the maize genotypes (at *P* ≤ 0.05, Figure 5), with the lowest proportion in the Southern European genotype Keravnos (3% of monomers) and highest in the Indian genotype 30 V 92 and the central European genotype Kentos (5.9% of monomers). No consistent relationship was detected between the mucilage polysaccharide composition of the genotypes and the vapor pressure deficit (VPD) of their agroecological zones of origin (Supplementary Table 1).

### Mucilage Exudation Amount and Saturation Water Content

**FIGURE 6:**
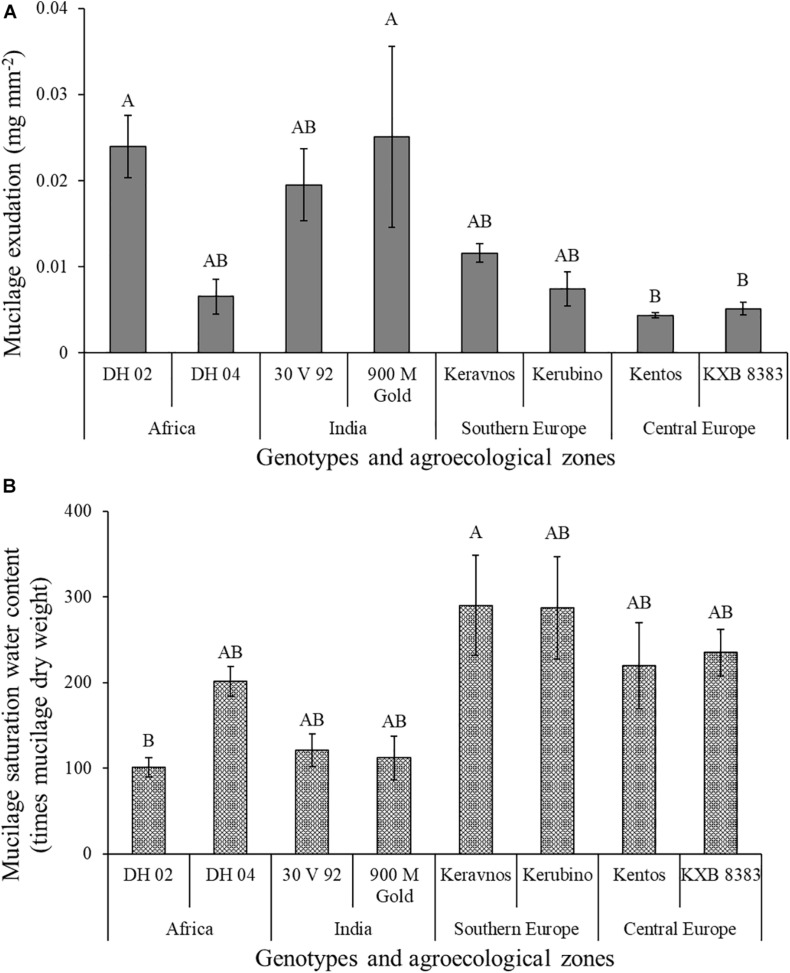
**(A)** Mucilage dry weight of the different maize genotypes normalized by root surface area. **(B)** Mucilage saturation water content of the maize genotypes. Different letters above the bars show significant differences between the respective water contents and exudation amounts (Tukey’s HSD, at *P* ≤ 0.05). Error bars indicate the standard error of the mean (*n* = 3).

**FIGURE 7:**
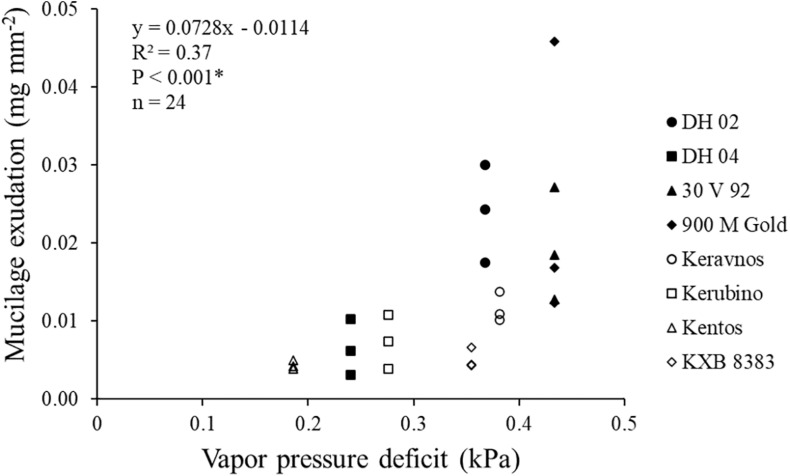
Relationship between the genotypes’ mucilage exudation and the vapor pressure deficit (VPD) of their agroecological zones (^∗^ = significant at *P* ≤ 0.05). The VPD for the region where the maize genotypes were grown for this study (Bavaria, Germany) was 0.19 kPa.

Mucilage exudation amount deviated significantly between the maize genotypes of contrasting origin investigated in this study (Table 2 and Figure 6A). The Indian genotype 900 M Gold and the Kenyan genotype DH 02 had a 135 and 125% higher mucilage production than the central European genotypes Kentos and KXB 8383. Mucilage exudation was positively correlated with the VPD of the agroecological zone for which the respective genotype was bred (Figure 7).

The maize mucilage absorbed on average 200 times its own dry weight in water. The mucilage saturation water content differed significantly between the maize genotypes. The mucilage of the southern European genotype Keravnos had the highest saturation water content, whereas the mucilage of the Kenyan genotype DH 02 had the lowest (Figure 6B). No significant relationship between the mucilage saturation water content of the genotypes and the VPD of their agroecological zones was found (Supplementary Figure 1). Moreover, no significant relationship between the mucilage saturation water content and its polysaccharide composition was detected (Supplementary Table 2).

## Discussion

### Mucilage Polysaccharide Composition

Galactose, fucose, mannose, and arabinose were the major neutral sugars in the nodal root mucilage of the maize genotypes, whereas xylose and glucose were shown to be of minor proportion. Sugar monomers liberated by acid hydrolysis are frequently used in soil science as biomarkers, assuming plants mainly produce pentose-rich polymers whereas microbes preferentially synthetize hexoses (Kögel-Knabner, 2002). This has led to proxies like the GM/AX ratio (galactose + mannose/arabinose + xylose) as a proxy for microbial vs. plant origin of soil organic matter (Spielvogel et al., 2016). Our study (Figure 3) demonstrates that mucilage sugar monomer composition would clearly fall into the microbial category and hence the utilization of such proxies, especially in the rhizosphere, needs to be used with caution (Gunina and Kuzyakov, 2015).

In previous studies, the polysaccharide composition of maize nodal root mucilage was similarly found to comprise fucose, galactose, arabinose, and mannose as the main neutral sugars (Van Deynze et al., 2018; Amicucci et al., 2019). The similarity in this pattern, especially the dominance of galactose, suggests that the polymeric structure of mucilage does not deviate greatly between genotypes from the polymeric structure proposed by Amicucci et al. (2019) i.e., a galactose backbone (most abundant monomer), which is highly fucosylated (2nd most abundant monomer) but also partially xylosylated, with further arabinan and mannoglucoronan branches. Van Deynze et al. (2018) and Amicucci et al. (2019) linked the polymeric structure of the nodal root mucilage to a functional role in attracting a specific plant-growth promoting microbial community. The general similarities in monomer composition suggest that this potentially linked function of nodal root mucilage to rapidly attract N-fixing microbial communities is present over a large set of genotypes and retained during progressive breeding from the landrace to the hybrids.

The proportion of xylose and glucose in the present study deviated from previous studies (Van Deynze et al., 2018; Amicucci et al., 2019). Whether glucosylation of the galactose backbone is partially replacing the xylosylation remains speculative and awaits confirmation by further LC-MS analysis in follow-up studies.

In terms of the uronic acids, glucuronic acid was the only uronic acid found in the mucilage of the maize genotypes, which is in agreement with the study of Van Deynze et al. (2018). Amicucci et al. (2019) found in addition low amounts of galacturonic acid (1%) and a much higher proportion of glucuronic acid (11.3%) in the nodal root mucilage. As the proportion of carboxylic groups (i.e., uronic acid monomers) accounts for the negative charge of the mucilage polymer, the landrace maize Sierra Mixe can be assumed to have much higher interactions via bivalent Ca^2+^ ion bonds (Brax et al., 2019). The ratio of specific Ca^2+^ ion bonds to unspecific electrostatic chain interactions was identified as a crucial parameter for hydration-dehydration kinetics as well as water flow through the mucilage, but only with biogels having considerably higher uronic acid content than the maize mucilage characterized in this study (Brax et al., 2019). Therefore, the relevance of the uronic acid-based Ca^2+^ interactions seems to be of rather minor importance for the nodal root mucilage of maize. Brax et al. (2019) found also a clear effect of the Ca^2+^ concentration on the gel properties, which might suggest an adaptation of the uronic acid content to the Ca^2+^ saturation of the soil solution. Although we do not have specific soil data available for the breeding stations of the genotypes presented here, it is most likely that the spectrum of soils covers calcareous as well as strongly decalcified acidic soils. Aboveground biogels, such as low-methoxy pectins, would show strongly contrasting rheological and hydraulic properties depending on the Ca^2+^ concentration of the soil solution. Nodal root mucilage of maize, with low uronic acid content but instead forming its 3D-structure with covalent as well as unspecific electrostatic interactions (Brax et al., 2019), will instead maintain similar structural and physicochemical features irrespective of the soil properties. However, this observation suggests that, although polygalacturonic acid (PGA) has been used in previous experiments to represent mucilage, PGA is in fact a rather weak analog of nodal root mucilage (Zickenrott et al., 2016; Brax et al., 2019; Ellerbrock et al., 2019).

Generally, differences in sugar monomer composition between the literature and our results need to be interpreted carefully, as they could either be related to genotype-specific differences or result from methodological differences. These differences could include plant growth stage at the time of mucilage collection, root type targeted for mucilage collection, mucilage collection method, hydrolysis time, hydrolysis temperature, purification methods, and derivatization methods (Chaboud and Rougier, 1984; Amicucci et al., 2019). Furthermore, a regulatory response of the plant to abiotic environmental conditions may have had significant influence on the mucilage composition. Conditions simulating the Sierre Mixe region of origin in Oaxaca (Mexico) (Amicucci et al., 2019), those in a greenhouse in Wisonsin (Van Deynze et al., 2018), and our field experiment in Bayreuth (Germany) would differ in temperature and humidity (VPD) as well as soil properties, with potential implications for the monomer composition (Van Deynze et al., 2018; Amicucci et al., 2019).

Our set of eight genotypes comprises a substantial breadth of breeders (KWS SAAT, Kenya Seed, Dekalb, and Pioneer Hi-Bred) and agroecological zones. We therefore expect that the variability of quantified sugar monomer composition, even between landraces and hybrids, would be much lower if a single standardized method were applied (Annette et al., 2020). Without detailed experimental comparisons of these methods, no final conclusion on the extent of possible methodological bias can be made. This calls for an inter-laboratory comparison of the relatively novel methodological approaches for sugar monomer analysis of mucilage and other biogels.

This study used one standardized methodological approach, and thus the identified differences in monomer composition between the eight genotypes, even if rather small, is robust. Nonetheless, the genotype-specific monosaccharide fingerprint of the mucilage could not be linked by a consistent relationship to the VPD in their agroecological zones of origin. Thus, further factors besides local climatic conditions might be responsible for the adaptation of the polysaccharide composition of the mucilage.

Compared to most previous studies, this experiment was not done in sterile hydroponic or greenhouse conditions on artificial substrates, but was a field experiment on natural soil and thus additional factors besides the plant genotype need to be considered. Ahmed et al. (2018a, b) have already shown that mucilage in soil may provide beneficial habitats for microbes. This was even more specifically demonstrated for nitrogen-fixing microorganisms in the aboveground mucilage on nodal roots (Triplett, 1996; Estrada et al., 2002; Montañez et al., 2009; Van Deynze et al., 2018; Amicucci et al., 2019). Evidence of mucilage C use for bacterial and fungal growth in soil (Ahmed et al., 2018a, b) as well as the specific mutualistic adaptation of defined glycosylhydrolases of microbial community members to the mucilage (Pozzo et al., 2018) suggest that the polysaccharide composition of the maize genotypes may have coevolved with beneficial soil microbial communities. Such microbiome-related carbohydrate adaptations would explain the absence of any correlation of monosaccharide composition with the VPD. However, more research with multiple factorial designs (e.g., full factorial designs growing genotypes on their native as well as contrasting soils) would be required to confirm mucilage-microbiome co-evolution. However, our data at least indicate that the nodal root mucilage polysaccharide composition may have a genetic basis, as the hybrids grown here under identical environmental conditions displayed genotype-specific polysaccharide patterns. Amicucci et al. (2019) observed significant differences neither between the polysaccharide linkages nor within the monosaccharide composition of the mucilage of Sierra Mixe maize grown under two different environmental conditions, which provides an indication for a potential genetic basis for the polysaccharide composition of nodal root mucilage in maize.

### Mucilage Exudation Amount and Saturation Water Content

The amount of mucilage exuded by maize genotypes from agroecological zones with higher VPD (Kenya and India) was considerably higher than the mucilage exudation of genotypes from zones with lower VPD (central Europe). There was a highly significant positive relationship between the mucilage exudation amount and the VPD of the genotypes’ agroecological zones of origin. Vapor pressure deficit (VPD) is an indirect measure of water stress for plants (Dai, 2013). Water depletion and thereby plant water stress increase with increasing VPD (Grossiord et al., 2020). The observed higher mucilage exudation in the Kenyan and Indian genotypes could be attributed to various factors. A very plausible explanation is the more frequent water deficiency in these regions, due to high temperatures and evapotranspiration (Mallick et al., 2007; Mutiga et al., 2010). Maize nodal roots are aboveground only for a short time and later enter the soil and become belowground roots, where they play a dominant role in water uptake (Ahmed et al., 2018d). Mucilage increases the rhizosphere water content (Young, 1995; Carminati et al., 2010). Higher mucilage exudation by the genotypes of the agroecological zones with higher VPD supports our hypothesis that mucilage exudation is inadvertently selected by plant breeding in water-stressed regions and displays an adaptive trait to drought—most likely to dry soils. This is consistent with the role of mucilage in sustaining water uptake and preventing root dehydration under dry conditions (Ahmed et al., 2018b). Furthermore, mucilage also reduces friction against the growing root in soil (Iijima et al., 2003). The maize genotypes from the semi-arid agroecological zones may exude high quantities of mucilage to ease seeking water in compacted soil layers. However, further research is required to experimentally verify this.

Another possible explanation can be a poor nutrient status in the soil of Kenya and India (Sharma et al., 2009; Mugo et al., 2020; Stewart et al., 2020), which represent our high-VPD agroecosystems. It has been shown that the landrace maize Sierra Mixe, originally from a nitrogen-depleted region in Mexico, gets a considerable amount of its nitrogen demand through biological nitrogen fixation by diazotrophs abundant in mucilage produced by its huge nodal roots (Van Deynze et al., 2018; Amicucci et al., 2019). It was also shown that maize nodal roots take up large amounts of nitrogen from the soil due to their length, high surface area, and a great number and density of lateral roots (Dechorgnat et al., 2018). Therefore, high mucilage exudation—especially of nodal roots—can also be an adaptive trait to nitrogen deficient soils, provided that breeding was not performed under optimal nitrogen supply.

Indications that mucilage exudation has probably a genetic component are also found in studies in Mexico, which showed that the ancient maize genotype teosinte (*Zea mays spp. mexicana*) exudes lower amounts of mucilage from its nodal roots compared to its domesticated landrace Sierra Mixe (Van Deynze et al., 2018). Our study indicates that genotype also governs mucilage exudation in commercial varieties.

The saturation water content of mucilage reported here (on average 200 times mucilage dry weight) is in agreement with previous studies, which fall in the range of 27–589 times dry weight (McCully and Boyer, 1997; Huang and Gutterman, 1999; Capitani et al., 2013). This very high water retention capability of mucilage is the basis for one of its key rhizosphere functions: providing a moist root environment, especially in dry and water-deficient conditions (Ahmed et al., 2018a). A multiple regression model was, however, not able to identify any significant relationships between the mucilage polysaccharide composition and its saturation water content. It seems possible that the mucilage water absorption capacity depends on other mucilage components such as protein and lipid content. This suggests further investigation to consider the potentially important functional roles of the minor chemical components of mucilage, the proteins and the lipids. Undoubtedly, we need to identify desirable biochemical and biophysical properties of mucilage for targeted breeding of beneficial mucilage composition and exudation amounts suited to defined agroecological zones.

## Conclusion

This study investigated the polysaccharide composition, exudation amount, and saturation water content of nodal root mucilage of maize genotypes from three continents. Galactose, fucose, mannose, and arabinose were the major neutral sugars in the mucilage of these maize genotypes. Xylose and glucose were minor components. Glucuronic acid was the sole uronic acid found in the mucilage of the maize genotypes. Significant differences were detected among the maize genotypes in the mucilage polysaccharide composition, exudation amount, and saturation water content. Mucilage exudation amount of maize might be linked to an adaptation to the climatic conditions of the agroecological zones for which the genotypes were bred. However, as previous studies suggested a strong coevolution between mucilage chemical composition and beneficial microbial communities, the nutrient status of the respective soils may have affected the polysaccharide’s monomer composition. This remains to be elucidated in further studies. This study suggests further experiments to include wild genotypes, landraces, and modern maize varieties and use these genotypes in multi-factorial field designs across agroecological zones globally. This may allow disentangling of the genotype × environment interactions that underlie mucilage composition and exudation. This would fundamentally extend our understanding of mucilage-related traits and may shed more light on their origin and selection.

## Data Availability Statement

The original contributions presented in the study are included in the article/Supplementary Material, further inquiries can be directed to the corresponding author/s.

## Author Contributions

MN and SR performed the lab experiments and parts of the field trial. CB supervised the lab experiments. AA and MC collected maize mucilage and arranged field-related works. KM-J assisted in manuscript preparation and scientific corrections. MD and MA designed and supervised the experiments. MN analyzed the data and prepared the manuscript draft. All authors read the final draft of the manuscript and shared their comments.

## Conflict of Interest

The authors declare that the research was conducted in the absence of any commercial or financial relationships that could be construed as a potential conflict of interest.
