# Supplementary material for: Mucilage Polysaccharide Composition and Exudation in Maize From Contrasting Climatic Regions
Source: Front Plant Sci. 2020 Dec 8;11:587610. doi: 10.3389/fpls.2020.587610 (PMC7752898; doi:10.3389/fpls.2020.587610)
Supplement: Supplementary file 1 [file Table_1.DOCX]

**Supplementary Table 1.** Relationship between the genotypes’ mucilage polysaccharide composition and the vapor pressure deficit (VPD) of their agroecological zones (linear regression, at P ≤ 0.05, n = 24).

| Dependent variable | Independent variable | df | Mean square | F | P-value | R^2^ |
| --- | --- | --- | --- | --- | --- | --- |
| Galactose  Fucose  Mannose  Glucose  Arabinose  Xylose  Glucuronic acid | VPD  VPD  VPD  VPD  VPD  VPD  VPD | 1  1  1  1  1  1  1 | 9.40  26.83  0.015  4.19  0.41  1.11  3.54 | 3.23  4.10  0.010  4.55  0.34  1.13  1.97 | 0.086 NS  0.055 NS  0.92 NS  0.055 NS  0.56 NS  0.29 NS  0.17 NS | 0.12  0.15  <0.0001  0.17  0.015  0.049  0.082 |

NS = not significant

**Supplementary Table 2.** Relationship between the genotypes’ mucilage saturation water content and its polysaccharide composition (multiple regression, at P ≤ 0.05, n = 24).

| Model/Variable | df | Mean square | F | P-value | R^2^ |
| --- | --- | --- | --- | --- | --- |
| Regression  Galactose  Fucose  Mannose  Glucose  Arabinose  Xylose  Glucuronic acid | 6  1  1  1  1  1  1  1 | 13034.97  6814.69  31.514  825.55  5841.56  886.38  4572.66  30882.46 | 1.92  0.80  0.00  0.09  0.68  0.10  0.53  4.18 | 0.13 NS  0.37 NS  0.87 NS  0.53 NS  0.12 NS  0.94 NS  0.35 NS  0.058 NS | 0.40  0.03  < 0.01  < 0.01  0.41  < 0.01  < 0.01  0.16 |

NS = not significant

**Supplementary Figure 1.** Relationship between the genotypes’ mucilage saturation water content and the vapor pressure deficit (VPD) of their agroecological zones (NS = not significant at P ≤ 0.05).
